# Rift Valley Fever Virus Infection among French Troops in Chad

**DOI:** 10.3201/eid0906.020647

**Published:** 2003-06

**Authors:** Jean Paul Durand, Michèle Bouloy, Laurent Richecoeur, Christophe Nicolas Peyrefitte, Hugues Tolou

**Affiliations:** * Tropical Medicine Institute of the French Army Medical Corps (IMTSSA), Marseille, France; † Institut Pasteur, Paris, France; ‡ 3ème Régiment d’Infanterie de Marine (RIMa), Vannes Cedex, France

**To the Editor:** During the rainy season every year, outbreaks of self-limiting nonmalarious febrile syndromes have occurred in French military troops on duty in Chad. To determine the cause of these syndromes, the Tropical Medicine Institute of the French Army Medical Corps implemented an arbovirus surveillance program in Marseille.

During summer 2001, we collected samples from 50 soldiers who had a febrile illness. All blood spot samples tested negative by enzyme-linked immunosorbent assay (ELISA) for certain antigens (i.e., dengue virus, West Nile virus, Chikungunya virus, and Wesselsbron virus). However, after co-culture of 31 peripheral blood lymphocyte samples with C6/36 and Vero cell lines collected in NDjamena, Chad, in August to September 2001, two strains of Rift Valley fever virus (RVFV) were isolated and identified by using indirect immunofluorescence with a specific mouse ascitic fluid and by using reverse transcriptase-polymerase chain reaction (RT-PCR) and sequencing. In retrospective testing, we found that all serum specimens tested by ELISA for RVFV-specific immunoglobulin (Ig) M and IgG were negative. The second serum samples from the two case-patients with these strains, collected 2 months later, were strongly positive (IgM 1/200,000; IgG 1/5,000).

Rift Valley fever, a febrile disease that affects livestock and humans, is transmitted by mosquitoes and caused by a virus (genus: *Phlebovirus,* family: *Bunyaviridae*) that can persist in nature in contaminated eggs. The virus was first isolated in Kenya in 1930 (*1*) and is endemic in the region. In Chad, the disease was first reported in 1967 at the same time as in Cameroon (*2*); no strain was isolated at that time. Since 1977, RVFV infection resulted in 600 deaths in Egypt (*3*), 300 in Mauritania in 1987 (*4*), and 200 in Saudi Arabia and Yemen (*5*,*6*) in 2000 to 2001.

To characterize these RVFV strains, parts of the three genome segments (L, M, and S) were amplified by using RT-PCR and sequenced as described (*7*,*8*). The figure shows the phylogenic tree constructed from the sequence of the region coding for NSs in the S segment, by using the neighbor-joining method implemented in Clustal W (version 1.6; available from: URL: http://www-igbmc.u-strasbg.fr/BioInfo/ClustalW/clustalw.html). The two strains identified in Chad are quite similar. They are located within the East/Central lineage established previously (*6*,*7*), which contains the virus that circulated in Madagascar (1991), Kenya (1997–1998), and Yemen and Saudi Arabia (2000–2001) (*9*,*10*). Sequencing of the region in the M and L segments led to the same clustering (not shown), suggesting that this virus did not evolve by reassortment. Determining the origin of the virus is difficult, but its genetic properties suggest that this strain has a Kenyan origin. Before this isolation, no RVFV strains from Chad had been genetically characterized. This strain may be endemic in this region of Central Africa, or the RVFV strain circulating in the Eastern countries may have been transported outside of the territory (which was likely the case in Yemen and Saudi Arabia in 2000) (*9*,*10*). Of the two case-patients, one soldier did not leave NDjamena during his 3-month tour of duty, whereas the other had been in contact with livestock in a flooded area before onset of symptoms. Contamination may have occurred through infected animals or mosquitoes, although sheep living in the area did not show any sign of disease (i.e., spontaneous abortions, deaths). The two cases we describe were self-limiting; however, deaths from this illness have been reported in nonepidemic settings in Central African Republic (*11*). Our data emphasize that healthcare providers should systematically consider Rift Valley fever as a diagnosis for febrile syndromes in persons returning from Africa, even in nonepidemic settings (*12*).

**Figure:**
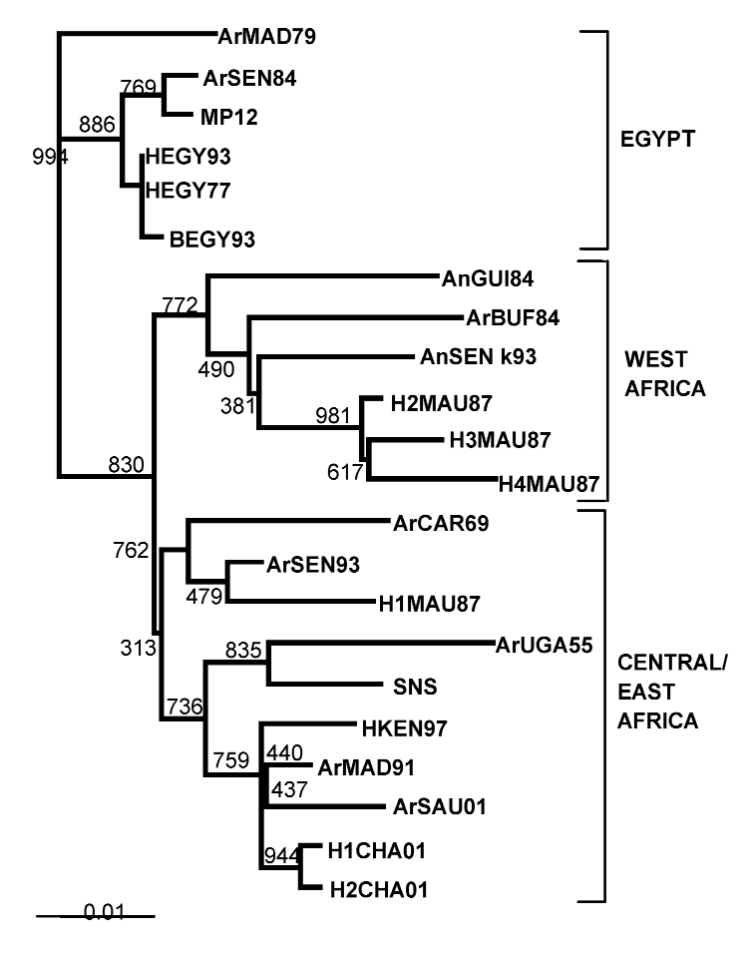
NSs-based phylogenetic tree of Rift Valley fever virus strains. Values indicate the bootstrap support of the nodes. Strains isolated in Chad are designated H1CHA01 and H2CHA01, according to the previous abbreviation guidelines (7,8). EMBL accession nos. AJ504997 and AJ504998. SNS, Smithburn strain. Branch lengths are proportional to the number of substitutions per site.
